# Smart Thermoresponsive Sol–Gel Formulation of Polyhexanide for Rapid and Painless Burn and Wound Management

**DOI:** 10.3390/polym17152079

**Published:** 2025-07-30

**Authors:** Levent Alparslan, Gülşah Torkay, Ayca Bal-Öztürk, Çinel Köksal Karayıldırım, Samet Özdemir

**Affiliations:** 1 Department of Pharmaceutical Technology, Faculty of Pharmacy, İstinye University, Istanbul 34396, Türkiye; 2 Stem Cell and Tissue Engineering Application and Research Center (ISUKOK), İstinye University, Istanbul 34396, Türkiye; gulsah.torkay@istinye.edu.tr (G.T.); aozturk@istinye.edu.tr (A.B.-Ö.); 3 Department of Stem Cell and Tissue Engineering, Institute of Graduate Education, İstinye University, Istanbul 34396, Türkiye; 4 Department of Analytical Chemistry, Faculty of Pharmacy, İstinye University, Istanbul 34396, Türkiye; 5 Department of Biology, Faculty of Science, Ege University, Izmir 35100, Türkiye; cinel.koksal@ege.edu.tr; 6 Department of Pharmaceutical Technology, Faculty of Pharmacy, İstanbul Health and Technology University, Istanbul 34445, Türkiye; samet.ozdemir@istun.edu.tr

**Keywords:** polyhexanide, sol–gel, wound treatment, burn care, thermoreversible gel, antiseptic, poloxamer 407

## Abstract

Traditional wound and burn treatments often fall short in balancing antimicrobial efficacy, patient comfort, and ease of application. This study introduces a novel, transparent, thermoresponsive sol–gel formulation incorporating polyhexamethylene biguanide (PHMB) for advanced topical therapy. Utilizing Poloxamer 407 as a biocompatible carrier, the formulation remains a sprayable liquid at room temperature and instantly gels upon contact with body temperature, enabling painless, pressure-free application on sensitive, injured skin. Comprehensive in vitro and in vivo evaluations confirmed the formulation’s broad-spectrum antimicrobial efficacy (≥5 log_10_ reduction in 30 s), high biocompatibility (viability > 70% in fibroblasts), non-irritancy (OECD 425-compliant), and physical stability across three months. Importantly, the formulation maintained fibroblast migration capacity—crucial for wound regeneration—while exhibiting rapid sol-to-gel transition at ~34 °C. These findings highlight the system’s potential as a next-generation wound dressing with enhanced user compliance, transparent monitoring capability, and rapid healing support, particularly in disaster or emergency scenarios.

## 1. Introduction

Wound and burn care remain critical areas in clinical practice, particularly due to the high risk of infection and delayed healing in compromised skin barriers. Polyhexanide (polyhexamethylene biguanide, PHMB) has gained significant attention in recent years as a broad-spectrum antiseptic with low cytotoxicity and potent biofilm-disrupting properties. Its effectiveness against multidrug-resistant organisms, such as *Staphylococcus aureus* (MRSA), *Pseudomonas aeruginosa*, and *Candida albicans*, makes it a promising alternative to traditional antimicrobials [1]. PHMB has been successfully incorporated into wound irrigation solutions, gels, and dressings, facilitating infection control and promoting tissue regeneration in both acute and chronic wounds [2,3]. Recent studies have demonstrated that PHMB-based formulations, especially in combination with betaine, significantly accelerate healing in superficial partial-thickness burns, with complete re-epithelialization observed within 15 days [4]. In pediatric burn care, PHMB application has shown to reduce healing time and hospital stays, highlighting its clinical utility [5]. Moreover, its ability to inhibit and disrupt biofilms enhances the success of advanced wound management strategies, including surgical reconstructions [6]. Collectively, these findings emphasize the importance of PHMB as a key component in next-generation wound and burn treatment modalities.

The integration of polyhexanide (PHMB) into sol–gel and thermoreversible hydrogel systems has emerged as a promising strategy in wound and burn care, offering enhanced therapeutic efficacy and patient comfort. Thermoreversible hydrogels, which transition from a liquid to a gel state at physiological temperatures, enable easy application and conformability to irregular wound surfaces, ensuring uniform drug distribution and sustained antimicrobial activity. A clinical study demonstrated that a thermoreversible PHMB-containing gel provided superior wound assessment capabilities and patient satisfaction compared to traditional treatments, highlighting its potential in clinical settings. Moreover, these hydrogels maintain a moist wound environment conducive to healing while minimizing the risk of maceration. Recent advancements in smart hydrogel technologies have focused on stimuli-responsive behaviors, such as temperature sensitivity, to facilitate controlled drug release and adapt to the dynamic wound microenvironment. The incorporation of PHMB into such systems not only enhances antimicrobial efficacy but also addresses challenges associated with biofilm formation and chronic wound infections. These innovations underscore the potential of sol–gel and thermoreversible PHMB formulations as effective interventions in modern wound management [7,8].

Poloxamer 407, a non-ionic triblock copolymer composed of poly (ethylene oxide)-poly (propylene oxide)-poly (ethylene oxide), is widely employed in pharmaceutical formulations due to its thermoresponsive gelation properties, biocompatibility, and ability to solubilize hydrophobic compounds [9]. At room temperature, it remains in a liquid state, but transitions to a gel at body temperature, making it particularly advantageous for wound and burn care applications, where ease of application and localized retention are critical [10]. It has been used as a base in sol–gel systems designed for controlled delivery of antimicrobial agents, including polyhexanide, enabling sustained contact with the wound bed without exerting mechanical stress on the tissue. Furthermore, multiple in vivo and clinical studies have confirmed its safety and non-irritant profile, even with repeated topical applications [11,12,13]. These properties support the use of Poloxamer 407 as an effective carrier in modern wound dressings, particularly when combined with antiseptics for enhanced therapeutic efficacy.

This study aims to optimize the thermoreversible transition of the formulation, enabling it to be applied as a solution at room temperature and to undergo gelation at body temperature. This approach is intended to minimize pain associated with contact during application, particularly in the treatment of burn injuries.

## 2. Materials and Methods

### 2.1. Materials

Polyhexanide (PHMB) was obtained from Germany as the commercial product Cosmocil PG^®^. Poloxamer 407 was purchased from Croda (Yorkshire, UK) under the trade name Synperonic^®^ PE/F127. Poloxamer 407 is a non-ionic triblock copolymer composed of poly (ethylene oxide)–poly (propylene oxide)–poly (ethylene oxide) (PEO–PPO–PEO) segments. The average molecular weight of Poloxamer 407 is approximately 12,600 Da, with an approximate PEO:PPO ratio of 70:30. Potassium dihydrogen phosphate was supplied by JT Baker (Deventer, The Netherlands). Sodium dihydrogen phosphate dihydrate, dipotassium hydrogen phosphate, and orthophosphoric acid were all procured from Merck (Darmstadt, Germany). Acetic acid was purchased from Riedel-de Haën (Seelze, Germany).

### 2.2. Preparation of the Formulation

A phosphate buffer solution at pH 7.4 was prepared according to the European Pharmacopoeia (Ph. Eur.) guidelines. Specifically, appropriate amounts of potassium dihydrogen phosphate (KH_2_PO_4_) and disodium hydrogen phosphate dihydrate (Na_2_HPO_4_·2H_2_O) were dissolved in purified water to yield 1000 mL of buffer with a final pH of 7.4. The pH was verified using a calibrated pH meter and adjusted if necessary using dilute hydrochloric acid or sodium hydroxide.

Cosmocil^®^ PG, a commercial polyhexamethylene biguanide (PHMB) preparation, was added to the buffer solution to achieve a final concentration of 0.2% *w*/*v*. The solution was stirred magnetically at room temperature until the PHMB was completely dispersed.

Following the incorporation of PHMB, Poloxamer 407 (PF 127) was slowly added to the system while stirring continuously to ensure uniform dispersion. The mixture was then stored at 4 °C for 24 h to allow for complete dissolution and gelation of the poloxamer, forming a thermoreversible sol–gel system.

### 2.3. Determination of Polyhexanide Content

The concentration of polyhexanide (PHMB) in the formulation was quantified using UV–visible spectrophotometric analysis (Jasco V-730 BIO, W. Yorkshire, UK). Measurements were performed at a wavelength of 235 nm, which corresponds to the characteristic absorbance maximum of PHMB. Prior to analysis, samples were appropriately diluted with phosphate buffer (pH 7.4) to ensure that the absorbance values fell within the linear range of the calibration curve.

The method demonstrated a limit of detection (LOD) of 20 mg/L, ensuring accurate quantification of PHMB even at low concentrations. Each sample was analyzed in triplicate, and the average value was used to calculate the final content.

### 2.4. Determination of Sol–Gel Transition Temperature

**Figure 1:**
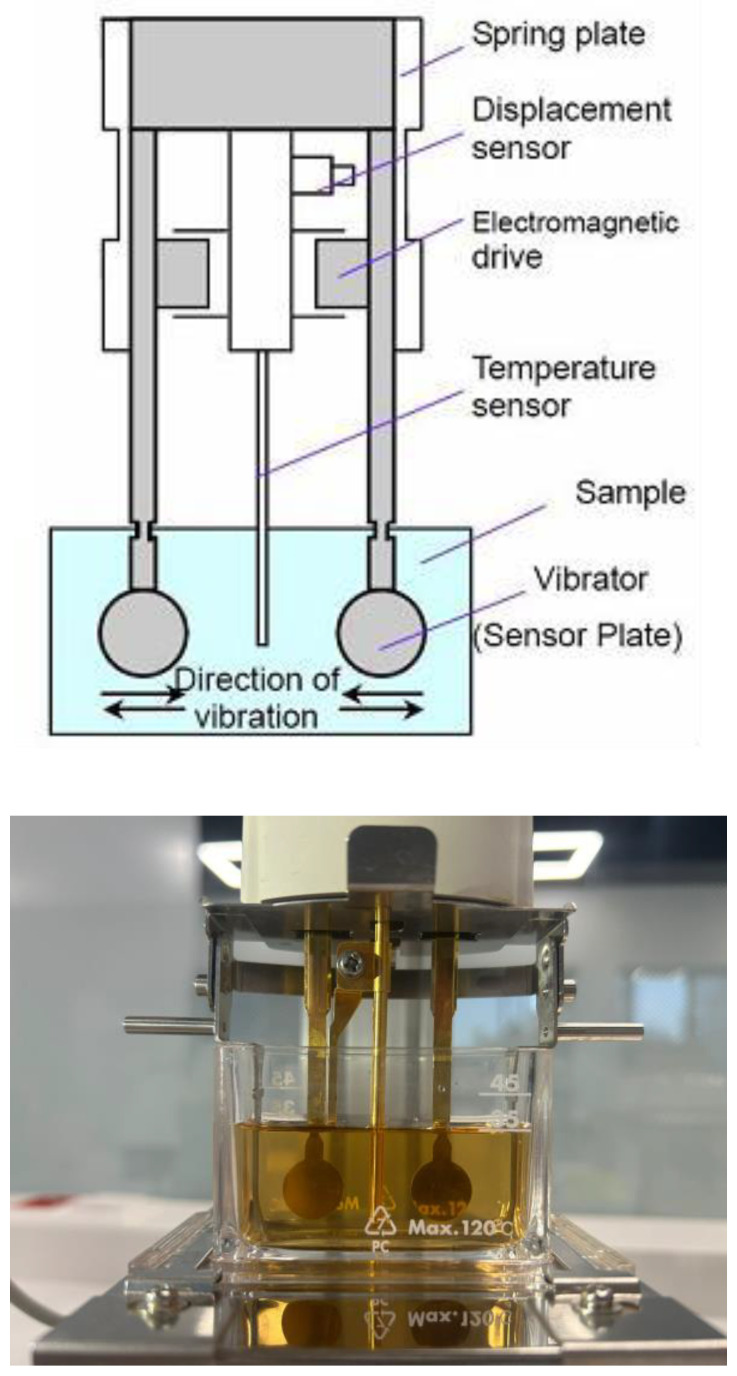
Schematic representation and photo of the tuning fork vibro-viscometer mechanism.

The sol–gel transition temperature of the formulation was determined using a vibro-viscometer (SV-10, A&D Instruments, Tokyo, Japan), based on the tuning fork vibration principle. This method allows for real-time and highly sensitive viscosity measurements by vibrating a pair of sensor plates immersed in the sample at a fixed frequency of 30 Hz. As the viscosity of the sample changes with temperature, the device measures the electrical power required to maintain the vibration, which correlates directly with the fluid’s viscosity. The system consists of two vibrating sensor plates (vibrators) driven by an electromagnetic motor. A displacement sensor and spring plate maintain vibrational resonance, while a temperature sensor enables precise thermal monitoring. The direction of vibration is horizontal, and the viscosity is calculated based on the energy required to sustain the vibration frequency (Figure 1).

Measurements were performed by gradually increasing the temperature of the formulation from room temperature to 42 °C. The viscosity profile was recorded continuously. A distinct increase in viscosity indicated the sol-to-gel transition point. The transition was characterized by a sharp rise in viscosity, corresponding to the gelation of the thermosensitive system. Upon subsequent cooling, the viscosity decreased and returned to its original value, demonstrating the reversible nature of the formulation’s thermal phase transition.

### 2.5. Microbiological Efficacy Evaluation

The antimicrobial activity of the sol–gel formulation was evaluated against five representative microbial strains: *Pseudomonas aeruginosa* (ATCC 9027), *Escherichia coli* (ATCC 8739), *Staphylococcus aureus* (ATCC 6538), *Enterococcus faecalis* (ATCC 29212), and *Candida albicans* (ATCC 10231). The testing protocol was conducted in accordance with TS EN 1276 [14] (for bactericidal activity) and TS EN 13624 [15] (for fungicidal activity) standards, which are commonly accepted for evaluating the hygienic effectiveness of antiseptic and disinfectant products.

The test method involved direct contact between the sol–gel formulation and microbial suspensions, followed by incubation for 30 s at room temperature. Subsequently, the number of surviving microorganisms was quantified by plating on suitable agar media and counting colony-forming units (CFUs).

### 2.6. In Vitro Cell Culture Studies

It is critical to conduct in vitro evaluations of developed pharmaceutical formulations. The human dermal fibroblast cell line (ATCC, CCD-1064Sk-CRL-2076) was utilized to assess the biocompatibility of the developed sol–gel formulations (PHMB-loaded and PHMB-free) and their impact on cell migration. Cells were cultivated in regular Dulbecco’s Modified Eagle Medium (DMEM; Thermo Fisher Scientific, Waltham, MA, USA) supplemented with 10% fetal bovine serum (FBS; Capricorn Scientific, Ebsdorfergrund, Germany) and 1% penicillin–streptomycin (Thermo Fisher Scientific, Waltham, MA, USA) and cultured in a 37 °C incubator with 5% CO_2_. Cells that achieved enough confluency were rinsed with Ca/Mg^2+^ free Dulbecco’s Phosphate-Buffered Saline (DPBS; Thermo Fisher Scientific, Waltham, MA, USA), then detached using trypsin (Thermo Fisher Scientific, Waltham, MA, USA), and seeded for specific tests.

#### 2.6.1. MTT Cytotoxicity Test

In order to determine the cytotoxicity of the developed sol–gel formulations and vehicle control (placebo) samples, which contained only polymer and no active ingredient, an MTT test was carried out. Samples were introduced into the medium at concentrations of 0.1%, 1%, and 5% by volume, and were initially sterilized by filtration through a 0.22 µm pore diameter polyethersulfone (PES) syringe filter prior to application. Cells were inoculated at a density of 8 × 10^3^ cells per well in 96-well plates and permitted to adhere overnight in the incubator. Once the cells adhered, the formulations prepared in the medium were administered to the cells at a volume of 100 µL per well. The control group was administered standard medium. After 24 h, representative photographs were taken from the wells, and then 10 µL of MTT solution (final concentration 0.5 mg/mL in culture medium) was introduced to the wells to assess cell viability. Following 4 h of incubation, the MTT solution was removed from the wells, and 100 µL of DMSO was used to dissolve the purple-violet formazan crystals. Following 5 min of shaking, absorbance at 570 nm was measured using an ELISA reader (BMG LABTECH SPECTROstar^®^ Nano, Ortenberg, Germany). Cell viability was determined using the following formula [16]:

(1) $$Cell\;Viability\;=\;\frac{{OD\;570}_{sample}}{{OD\;570}_{control}}\;\times 100$$

Here, OD 570_sample_ is the absorbance of the sol–gel formulations and placebo sample-treated groups at 570 nm; OD 570_control_ is the absorbance of the control group at 570 nm.

#### 2.6.2. Cell Migration Test

To assess how the developed PHMB-loaded and PHMB-free sol–gel formulations affected cell migration, an in vitro scratch test was conducted. For this test, the group with the highest percentage viability compared to the control in MTT data (as 0.1%) was used. The formulations were produced identically, but with 5% FBS instead of 10%. A total of 80 × 10^3^ cells per well were seeded in 24-well plates, and after confluence, a gap was induced in the monolayer using a sterile 200 μL micropipette tip to mimic a wound. Cellular debris and floating cells were eliminated through washing with PBS. The prepared media were administered to the wells. The control groups received a standard medium including 5% FBS. The plate was incubated at 37 °C in a 5% CO_2_ atmosphere. Images were captured using an inverted microscope (Zeiss Observer Z1 with CO_2_ Incubation System, Carl Zeiss, Jena, Germany) at certain time intervals and coordinates [17].

### 2.7. Skin Irritation Test

The skin irritation potential of the sol–gel formulation was assessed in compliance with the OECD Test Guideline No. 404 (Acute Dermal Irritation/Corrosion). The study was conducted using healthy New Zealand albino rabbits, each weighing between 2.5 and 3.5 kg, under controlled laboratory conditions with appropriate animal care and housing procedures.

Prior to application, the dorsal area of each rabbit was carefully shaved (approximately 6 cm^2^). A fixed amount of the test formulation was applied topically to the exposed skin and covered with a semi-occlusive dressing for a period of 4 h. After removal of the dressing, the test sites were observed and evaluated at 1, 24, 48, and 72 h for any signs of erythema (redness) or edema (swelling) according to the Draize scoring system.

This study was approved by the Local Ethical Committee of Animal Experiments at Ege University (02.08.2012, 2013-013). Ethical guidelines for the investigation of experimental pain in conscious animals were considered all in vivo experiments [18]. Healthy young adult White New Zealand rabbits were obtained from Ege University (Izmir, Turkey). The animals were housed under specific controlled pathogen-free conditions consisting of a 12 h light/dark cycle, a temperature of 20 °C, a relative humidity of 50%, and free access to water and food.

At the beginning of the study, the dorsal skin of rabbits (1 male, 1 female) under anesthesia was removed. Then, 0.5 mL of sol–gel was applied to the 6 cm^2^ of dorsal skin area and covered with a gauze patch. The 14-day observation period was sufficient to evaluate the effects of the gel [19,20].

### 2.8. Quality Control of the Sol–Gel

The final formulation was evaluated for its physicochemical properties, including density (calibrated pycnometer), pH and electrical conductivity (Mettler Toledo, Hertfordshire, UK), and osmolality (Knauer-Semi-Micro Osmometer, Berlin, Germany), using standard analytical procedures. All measurements were performed at room temperature and recorded in triplicate to ensure reproducibility. In addition, particle size distribution and homogeneity were assessed using dynamic light scattering (DLS) with a Malvern Zetasizer Nano ZS (Malvern Panalytical, Worcestershire, UK). The instrument calculates the hydrodynamic diameter based on the time-dependent fluctuations in light scattering intensity caused by Brownian motion. The particle size was derived using the Stokes–Einstein equation, assuming spherical and non-interacting particles suspended in a Newtonian fluid. Measurements were performed at 25 °C using disposable sizing cuvettes with a path length of 10 mm. The sample, composed of PHMB-loaded sol–gel dispersed in Poloxamer 407 aqueous solution, was equilibrated prior to analysis. The instrument was operated with a refractive index (RI) of 1.360 and a viscosity value of 105 cP, as determined for the gel matrix.

## 3. Results

### 3.1. Physicochemical Properties

The developed sol–gel formulation exhibited a pH value of 7.36 ± 0.02, which is appropriate for maintaining skin integrity and supporting antimicrobial efficacy. The density was measured as 1.028 ± 0.001 g/mL, confirming the homogeneity and stability of the formulation matrix. Osmolality measurements revealed a value of 380 ± 0.0 mOsm/kg, indicating a slightly hypertonic nature suitable for topical applications. Electrical conductivity was determined as 1935 ± 0.6 µS/cm, suggesting good ionic strength of the formulation for consistent performance. The refractive index values were recorded between 1.330 and 1.358, indicating the optical clarity and uniformity of the preparation.

### 3.2. Polyhexanide Content Determination

**Figure 2:**
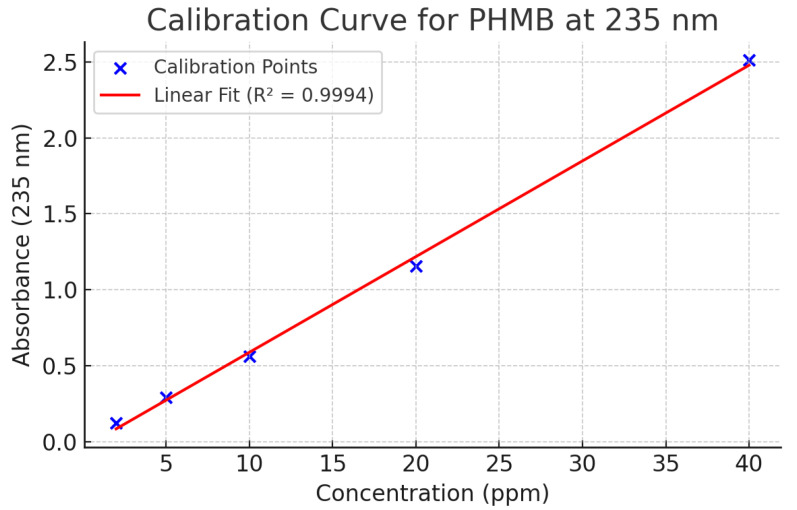
Calibration curve of PHMB at 235 nm.

Spectrophotometric analysis at 235 nm confirmed that the polyhexanide (PHMB) content remained within ±15% of the target concentration (0.2% *w*/*v*) throughout the formulation and storage process (Table 1). The method demonstrated high sensitivity, with a limit of detection (LOD) of 20 mg/L (Figure 2). All tested samples maintained consistent active ingredient concentrations, validating the formulation’s chemical stability and reproducibility.

### 3.3. Sol–Gel Transition Temperature

**Figure 3:**
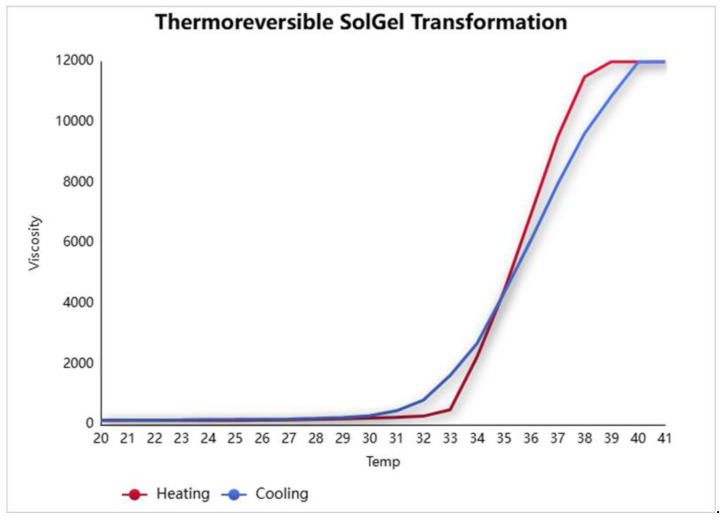
Transition of the fluidity of the sol–gel formulation according to temperature.

**Figure 4:**
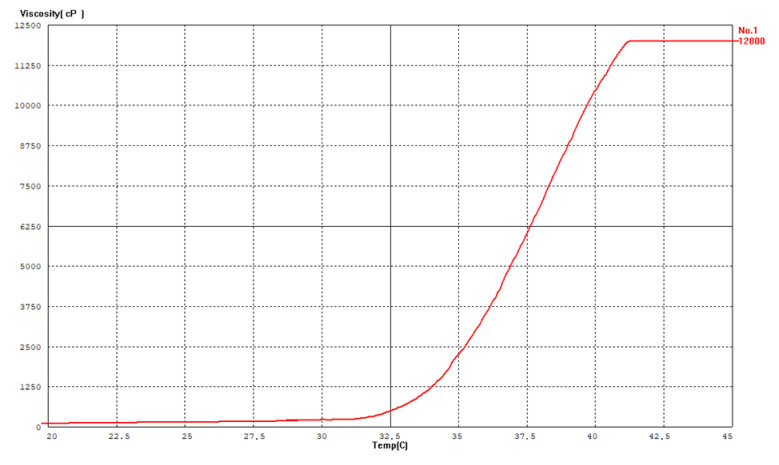
Measurement of sol–gel phase transition in the vibroviscometer.

**Figure 5:**
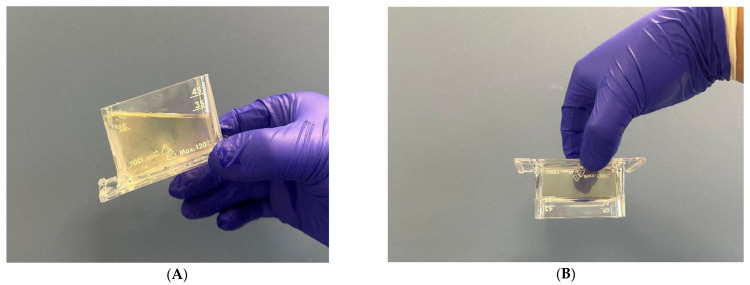
(**A**) The fluidity of the formula at room temperature. (**B**) Before measurement and its gel state at 40 °C.

The sol–gel transition behavior was monitored using a vibro-viscometer (SV-10), based on the tuning fork vibration principle. Upon gradual heating from room temperature to 42 °C, a marked increase in viscosity was observed at approximately 33 °C, indicating the sol-to-gel transition (Figure 3 and Figure 4). Cooling the system caused a reduction in viscosity, confirming the reversible thermoresponsive behavior of the formulation (Figure 3). This property is critical for ease of application and ensures that gelation occurs only upon contact with body temperature, enhancing wound surface coverage (Figure 5).

**Figure 6:**
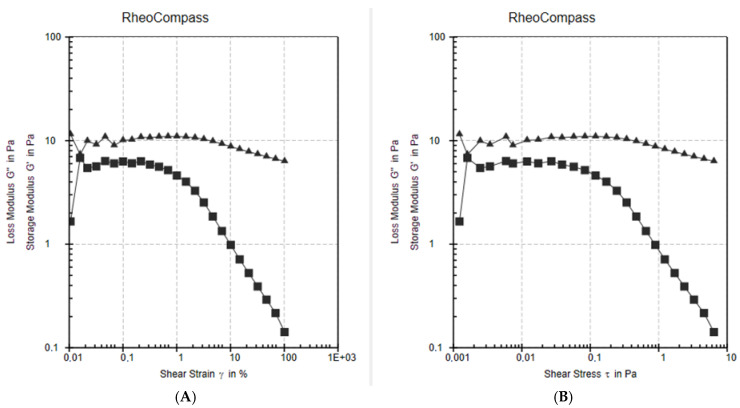
Amplitude sweep rheological analysis of the PHMB-loaded sol–gel formulation at 25 °C, showing the variation of storage modulus (G′) and loss modulus (G″) as a function of (**A**) shear strain (%) and (**B**) shear stress (Pa).

The sol–gel behavior was also measured using a rotational rheometer. Share strain and amplitude sweep tests were performed (Anton Paar MCR series with RheoCompass^®^ 1.25 software) at **25 °C**, simulating **room temperature** conditions. The results are presented in Figure 6, showing both storage modulus (G′) and loss modulus (G″) as a function of **shear strain (%)** and **shear stress (Pa)**.

At **25 °C**, G″ (loss modulus) is consistently **greater than G′** (storage modulus), indicating that the formulation exists in a **predominantly liquid-like sol state**, which supports its **sprayable** behavior at room temperature.

The **G′/G**″ **crossover point**—the theoretical sol–gel transition—was not reached at this temperature, further verifying that gelation does **not** occur below physiological temperatures.

### 3.4. Microbiological Efficacy

The antimicrobial activity evaluation against *Pseudomonas aeruginosa*, *Escherichia coli*, *Staphylococcus aureus*, *Enterococcus faecalis*, and *Candida albicans* demonstrated a ≥5 log_10_ reduction in CFU within 30 s of exposure. The testing procedures, compliant with the TS EN 1276 and TS EN 13624 standards, confirmed that the sol–gel formulation achieved rapid and significant microbial load reduction, thus meeting the efficacy requirements for antiseptic products intended for wound and burn care.

The validation data and CFU counts for *Pseudomonas aeruginosa, Escherichia coli, Staphylococcus aureus*, and *Enterococcus faecalis* were obtained, including the calculated log reductions (all exceeding 5 log_10_), with raw CFU values and standard deviation ranges across the replicates (*n* = 3).

The PHMB sol–gel formulation achieved a >5 log_10_ CFU reduction within 30 s for all bacterial strains tested, meeting the EN 1276 standard under clean conditions. Detailed CFU counts, validation data, and log reduction calculations are presented in Table 2, Table 3, Table 4 and Table 5.

### 3.5. In Vitro Cell Culture Results 

**Figure 7:**
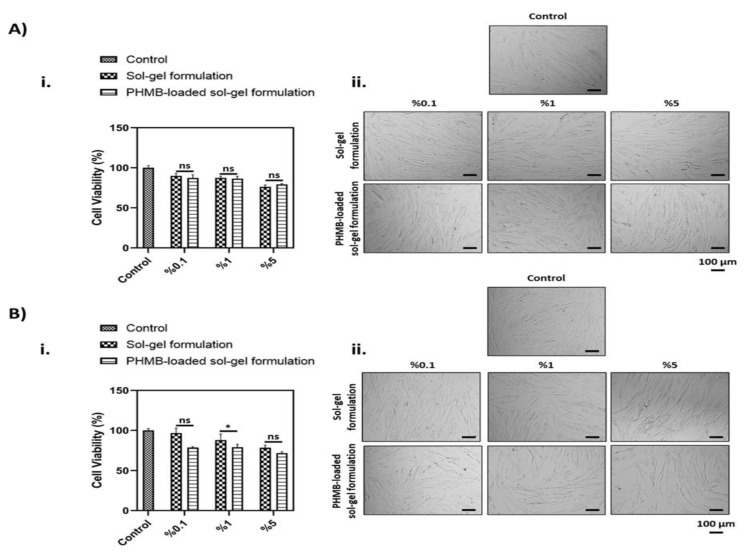
Evaluation of the effects of the developed PHMB-free and PHMB-loaded sol–gel formulation on dermal fibroblasts. MTT assay results after (**A**) 24 h and (**B**) 48 h of exposure to control and sample groups. (**i**) Percentage viability values and (**ii**) Representative images of cell morphology (scale bar: 100 µm). Statistical analysis was performed using one-way ANOVA (data are presented as mean ± standard deviation, *n* = 5). * *p* < 0.05.

The % viability values obtained after 24 h and 48 h of incubation, as shown in Figure 7, reflect the cytotoxic effects of the thermoresponsive PHMB-free and PHMB-loaded sol–gel formulations compared to the control group. Statistical analysis revealed that cell viability significantly decreased in all sample groups compared to the control. Specifically, the % viability values after 24 h of incubation were recorded as 100.02 ± 2.67 for the control group. For the 0.1% concentration, viability values were 89.99 ± 3.03 (placebo, PHMB-free sol–gel formulation) and 87.29 ± 3.97 (PHMB-loaded sol–gel formulation). At the 1% concentration, the values were 87.48 ± 2.05 (placebo) and 86.40 ± 2.98 (PHMB-loaded sol–gel formulation). For the 5% concentration, viability values declined further to 76.17 ± 2.39 (placebo) and 79.18 ± 1.02 (PHMB-loaded sol–gel formulation) (Figure 7A(i)). Based on the generally accepted threshold of 70% viability for non-cytotoxic materials [21], all groups can be considered non-toxic. To further substantiate these findings, the cellular morphology was evaluated prior to the MTT assay at the 24 h time point (Figure 7A(ii)). Morphological examination revealed comparable cell density and structure to the control group across all groups, where signs of cytotoxicity, such as cell detachment and rounded morphology, were not observed. Similarly, the results obtained after 48 h of exposure are presented in Figure 7B. Consistent with the 24 h findings, the percentage of viable cells remained above the critical 70% threshold at 48 h, indicating that all tested formulations maintained acceptable biocompatibility over extended exposure. The cell viability in the control group was calculated as 100.05 ± 2.23. In the placebo (PHMB-free) sol–gel formulation, the viability rates were determined as 96.73 ± 6.13%, 87.78 ± 7.62%, and 78.35 ± 3.04% for the 0.1%, 1%, and 5% concentrations, respectively. In the PHMB-loaded sol–gel formulation, these values were 78.62 ± 0.93%, 78.93 ± 3.87%, and 71.84 ± 1.70% at the same concentrations (Figure 7B(i)). The noted decrease in viability at higher concentrations, especially in the PHMB-loaded groups, aligns with the current literature indicating the dose-dependent cytotoxicity of PHMB [8]. These data highlight the significance of concentration and exposure time in assessing cytocompatibility. Despite a slight decrease in metabolic activity, the examination of cell morphologies revealed that the sol–gel groups seemed like the healthy control group, indicating a cytostatic effect rather than a lethal effect.

Our findings are consistent with prior studies emphasizing the significance of formulation strategies in mitigating PHMB-induced cytotoxicity without compromising antimicrobial efficacy. For example it was demonstrated that oil-in-water emulsions and liposomal systems incorporating egg yolk phosphatidylcholine effectively protected mammalian fibroblasts from PHMB-mediated toxicity while retaining bactericidal activity [22]. In a similar manner, our thermos-responsive sol–gel formulation likely acted as a biocompatible delivery matrix, enabling the controlled release and spatial confinement of PHMB. This effect was particularly evident at the 0.1% concentration, where no adverse impact on cell viability was observed.

**Figure 8:**
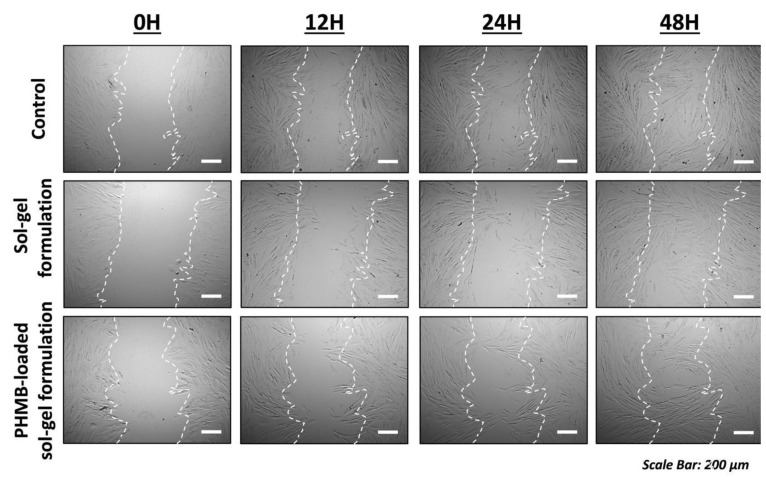
Evaluation of dermal fibroblast migration after exposure to PHMB-free and PHMB-loaded sol–gel formulation. Representative images of scratch closure at different time intervals (0, 12, 24, and 48 h) are shown for the control and sample groups. The closure of the scratch area was comparable between the control and sol–gel formulation groups, indicating that the developed sol–gel formulations did not impair cell migration (scale bar: 200 um).

The in vitro scratch assay was conducted exclusively with the 0.1% concentration to assess the impact of the developed PHMB-loaded sol–gel formulation on dermal fibroblast migration. Figure 8 illustrates that the closure of the scratch area in the extract-treated groups was similar to that in the control group, suggesting that cell migration was not negatively impacted. The MTT assay indicates a slight reduction in mitochondrial activity after 24 h of exposure to the samples, with viability remaining over 70%; however, these findings do not inherently reflect a diminished migratory capacity. The MTT assay largely indicates mitochondrial metabolic activity, while the scratch assay evaluates collective cell movement. Additionally, the scratch assay was conducted under diminished serum conditions (5% FBS), which may amplify any possible inhibitory effects on cell migration. Notwithstanding this more rigorous environment, no postponement in wound closure was noted, further corroborating the biocompatibility of the sol–gel formulations at low concentrations. Collectively, these findings indicate that the developed PHMB-loaded sol–gel formulation, at a concentration of 0.1%, does not adversely affect fibroblast migration, a critical characteristic for wound healing and regeneration applications.

### 3.6. Skin Irritation Evaluation

The acute dermal irritation of sol–gel was evaluated using an in vivo test method OECD No 404 using rabbits.

The skin irritation potential of the formulation was assessed using New Zealand albino rabbits in accordance with OECD Test Guideline No. 404. Throughout the 72 h observation period following topical application, no signs of erythema or edema were detected. According to the Primer Irritation Index (PII), no adverse clinical effect or skin damage to the epidermis (irritation, corrosion etch) were observed. These results support the safety of the sol–gel system for topical use on wounded or sensitive skin areas.

**Figure 9:**
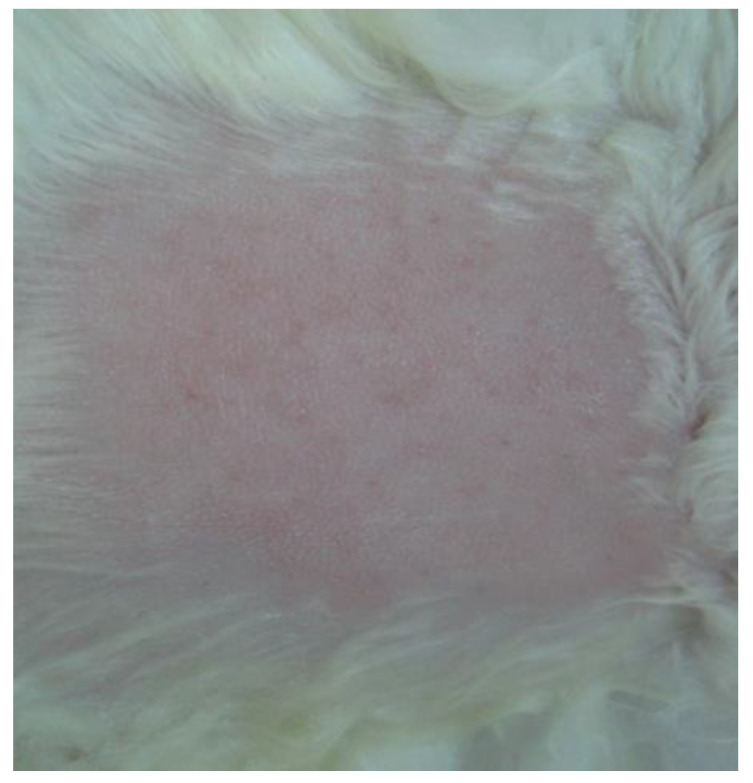
Representative image of rabbit dorsal skin at 72 h post-application, showing absence of erythema or edema.

- A **quantitative scoring table** (Table 6) showing the Draize scale-based erythema and edema scores at 1, 24, and 72 h for both male and female rabbits. All observations recorded were “N.O.” (not observed), corresponding to a score of 0.
- Based on these results, the **calculated Primary Irritation Index (PII)** is confirmed as **0**, indicating that the formulation is **non-irritant** under the applied conditions.
- A **representative image of the dorsal skin region** at 72 h has also been added as Figure 9, showing no signs of erythema, swelling, or skin damage after gel application.

We acknowledge that due to the retrospective nature of this test, the data set is limited to available records. However, the core safety results are intact and clearly demonstrate the dermal tolerability of the PHMB-loaded sol–gel formulation.

### 3.7. Characterization of the Sol–Gel

The formulation maintained its physicochemical and antimicrobial properties during three months of refrigerated storage, with consistent sol–gel transition behavior after repeated temperature cycling (Table 7).

**Figure 10:**
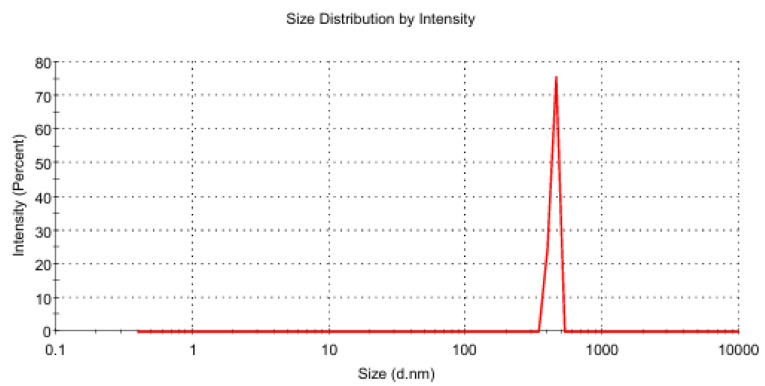
Intensity-based particle size distribution of the PHMB-loaded sol–gel formulation measured by dynamic light scattering (DLS).

The particle size analysis of the optimized PHMB-loaded sol–gel formulation, conducted via dynamic light scattering (DLS), revealed a Z-average diameter of 318.0 nm and an exceptionally low polydispersity index (PdI = 0.056), indicating a highly monodisperse and uniform particle population (Figure 10). The count rate of 372.6 kcps supports the reliability of the measurement, while the system’s viscosity of 105 cP at 25 °C aligns well with the expected rheological profile of a thermoreversible gel. These findings suggest a well-structured and physically stable formulation, suitable for topical application in wound and burn care, offering consistent drug release and favorable mechanical behavior upon administration.

## 4. Discussion

The development of an effective wound care system requires a delicate balance between antimicrobial efficacy, patient safety, ease of application, and maintenance of a moist wound environment. In this study, a novel PHMB-loaded thermoresponsive sol–gel formulation was successfully developed and characterized for wound and burn treatment applications.

PHMB is a cationic polymeric biguanide that acts primarily through electrostatic interactions with negatively charged bacterial cell membranes, leading to the following:

- The disruption of membrane integrity, causing leakage of cytoplasmic contents;
- The condensation of bacterial DNA, inhibiting replication and transcription;
- Biofilm disruption, by penetrating and destabilizing extracellular polymeric substances (EPS).

These mechanisms collectively result in bactericidal activity within seconds to minutes, even against multidrug-resistant organisms, such as *Pseudomonas aeruginosa*, *Staphylococcus aureus*, and *Candida albicans*.

In the context of a thermoresponsive sol–gel system, the inclusion of PHMB enhances local antimicrobial activity, while the gel matrix (Poloxamer 407) may provide prolonged retention, controlled release, and minimal systemic exposure, improving therapeutic efficacy and biocompatibility [22,23,24].

PHMB exhibits broad-spectrum bactericidal and fungicidal effects, with demonstrated activity against biofilm-forming organisms (e.g., *P. aeruginosa*, *S. aureus*, *C. albicans*) at low concentrations and in short contact times (30–60 s). Compared to povidone–iodine, which is effective but often cytotoxic at therapeutic concentrations, PHMB demonstrates significantly lower cytotoxicity while maintaining efficacy. Chlorhexidine has potent antimicrobial action but is less effective against fungi and can exhibit local toxicity, especially in higher concentrations. Triclosan use has declined due to resistance and regulatory concerns. Octenidine has efficacy similar to PHMB but can show more local irritation in open wounds. Studies have shown that PHMB has a favorable biocompatibility index, especially when compared to povidone–iodine and chlorhexidine, which often impair fibroblast proliferation and wound closure. Our MTT data further support that the developed PHMB-loaded sol–gel maintained >70% cell viability, aligning with the literature showing PHMB as a safer antiseptic for regenerative processes [7,25].

Our formulation supports fibroblast migration, a critical factor in wound regeneration, as confirmed by the in vitro scratch assay.

The physicochemical characterization demonstrated that the formulation maintained a neutral pH, optimal density, osmolarity, and excellent physicochemical stability over time. These parameters are critical for ensuring compatibility with the wounded tissue and for preserving the functional integrity of the active ingredient.

Our findings align with previous studies reporting the advantages of using poloxamer-based thermogelling systems for topical delivery. For example, Alparslan et al. demonstrated that thermoreversible sol–gels based on Poloxamer 407 could significantly enhance mucosal residence time and controlled drug release in nasal applications. Similarly, in wound care, a sol–gel matrix offers superior coverage, adaptation to wound topography, and controlled release properties, compared to traditional semi-solid dosage forms.

The microbiological results were particularly promising, showing a ≥5 log_10_ reduction in major wound pathogens (*Pseudomonas aeruginosa*, *Escherichia coli*, *Staphylococcus aureus*, *Enterococcus faecalis*, and *Candida albicans*) within just 30 s. Our formulation, however, offers an additional advantage of a highly reversible sol–gel phase transition at physiological temperatures, ensuring easy application as a liquid and in situ gelation at the wound site.

Moreover, the skin irritation test confirmed that the formulation was non-irritant, supporting its safety profile for repeated topical use. Importantly, the sol–gel system developed in this study exhibited excellent thermoreversibility and stability. Unlike conventional creams or ointments that might stain, leak, or require complex removal procedures, our transparent, reversible gel allows for easy visualization of the wound bed and can be gently removed without trauma—an important advantage also emphasized in previous clinical evaluations.

Overall, this study presents a significant innovation by combining the strong antimicrobial properties of PHMB with the intelligent behavior of a poloxamer-based sol–gel matrix. Compared to existing formulations, the developed system offers rapid antimicrobial action, excellent skin compatibility, enhanced patient compliance, and improved wound assessment visibility, suggesting a promising therapeutic option for the management of acute and chronic wounds. The formulation was precisely engineered to remain in liquid form at room temperature (~25 °C) and to gel rapidly upon contact with skin (~33 °C), as confirmed by both vibroviscosity and rheometry data. This narrow thermal window ensures that the formulation can be sprayed without pressure yet gels instantly when applied to the body, forming a transparent, non-dripping film that adheres to the wound site. In contrast to many semi-solid wound dressings or gels, which require manual spreading, gauze contact, or painful removal, our system minimizes mechanical irritation and enhances patient compliance, especially in pediatric or mass casualty settings. Furthermore, the formulation was developed as a sprayable pharmaceutical form, which is rarely reported in the literature for PHMB-based thermogels. This dosage form enables uniform coverage, reduced contamination risk, and ease of use in field or outpatient settings, which is an advantage over syringe- or spatula-applied thermogels.

While several studies have demonstrated the benefits of PHMB in gel systems, these have often utilized pre-formed gels or multi-step application protocols [7]. Our study presents a single-phase, ready-to-use sol–gel, optimized for real-time conversion without external manipulation, enhancing speed and practicality.

These application-based innovations, although not focused on novel actives or stability mechanisms, present a meaningful advancement in terms of usability and clinical handling, which we believe will contribute to better wound management protocols in acute and emergency care.

Future work will focus on long-term clinical evaluations, in vivo wound healing studies, and biofilm disruption assays to further substantiate the clinical benefits of this thermoresponsive PHMB-loaded sol–gel platform.

## 5. Conclusions

A novel PHMB-loaded thermoresponsive sol–gel was successfully developed, demonstrating excellent physicochemical stability, potent antimicrobial activity, biocompatibility, and thermoreversible behavior. These results position the formulation as a promising candidate for advanced wound and burn care management. Further clinical studies are warranted to evaluate its long-term efficacy and patient outcomes.

## Figures and Tables

**Table 1:** PHMB concentrations versus absorbance.

Standard Name	PHMB (ppm)	Absorbance (235 nm)
PHMB 2 ppm	2.0	0.125
PHMB 5 ppm	5.0	0.291
PHMB 10 ppm	10.0	0.562
PHMB 20 ppm	20.0	1.156
PHMB 40 ppm	40.0	2.511

**Table 2:** Validation and controls of *P. aeruginosa* treated by PHMB formulation.

Validation Suspension (*Nv0*)		A			B			C	
C1_V_	92	C1_V_	84		C1_V_	73		C1_V_	99
C2_V_	70	C2_V_	68		C2_V_	85		C2_V_	75
$\bar{x}\;$= 81		$\bar{x}$ = 76			$\bar{x}$ = 79			$\bar{x}$ = 87	
Suspension of Test and Test (N and N_0_)		*N*	V_C_		$\overset{¯}{\underset{¯}{x}}$ wm = ×10^6^ log_N_ = 8.36 N_0_ = N/10 log_No_ = 7.36 7.17 ≤ log_No_ ≤ 7.70				
		10^−6^	216	229					
		10^−7^	21	37					
PHMB Concentration (%)		C1_V_	C2_V_		$\mathrm{Na}\;=\;\underline{x}\;$× 10		Log_Na_	Log_R_	Sec.
0.2		0	1		<140		<2.15	>5.21	30

**Table 3:** Validation and controls of *E. coli* treated by PHMB formulation.

Validation Suspension (*Nv0*)		A			B			C	
C1_V_	94	C1_V_	73		C1_V_	92		C1_V_	78
C2_V_	68	C2_V_	85		C2_V_	88		C2_V_	80
$\bar{x}$ = 81		$\bar{x}$ = 79			$\bar{x}$ = 90			$\bar{x}$ = 79	
Suspension of Test and Test (N and N_0_)		*N*	V_C_		$\underline{x}$ wm = ×10^6^ log_N_ = 8.38 N_0_ = N/10 log_No_ = 7.38 7.17 ≤ log_No_ ≤ 7.70				
		10^−6^	221	247					
		10^−7^	19	26					
PHMB Concentration (%)		C1_V_	C2_V_		$\mathrm{Na}\;=\;\bar{x}$ × 10		Log_Na_	Log_R_	Sec.
0.2		0	0		<140		<2.15	>5.23	30

**Table 4:** Validation and controls of S. aureus treated by PHMB formulation.

Validation Suspension (*Nv0*)		A			B			C	
C1_V_	97	C1_V_	88		C1_V_	79		C1_V_	73
C2_V_	81	C2_V_	92		C2_V_	87		C2_V_	65
$\bar{x}$ = 89		$\bar{x}$ = 90			$\bar{x}$ = 83			$\bar{x}$ = 69	
Suspension of Test and Test (N and N_0_)		*N*	V_C_		$\bar{x}$ wm = ×10^6^ log_N_ = 8.39 N_0_ = N/10 log_No_ = 7.39 7.17 ≤ log_No_ ≤ 7.70				
		10^−6^	229	247					
		10^−7^	28	42					
PHMB Concentration (%)		C1_V_	C2_V_		$\mathrm{Na}\;=\;\bar{x}$ × 10		log_Na_	log_R_	Sec.
0.2		1	1		<140		<2.15	>5.24	30

**Table 5:** Validation and controls of *E. faecalis* treated by PHMB formulation.

Validation Suspension (*Nv0*)		A			B			C	
C1_V_	98	C1_V_	83		C1_V_	94		C1_V_	88
C2_V_	276	C2_V_	75		C2_V_	72		C2_V_	96
$\bar{x}$ = 87		$\bar{x}$ = 79			$\bar{x}$ = 83			$\bar{x}$ = 92	
Suspension of Test and Test (N and N_0_)		*N*	V_C_		$\bar{x}$ wm = ×10^6^ log_N_ = 8.32 N_0_ = N/10 log_No_ = 7.32 7.17 ≤ log_No_ ≤ 7.70				
		10^−6^	196	217					
		10^−7^	19	28					
PHMB Concentration (%)		C1_V_	C2_V_		$\mathrm{Na}\;=\;\bar{x}\;$× 10		log_Na_	log_R_	Sec.
0.2		1	0		<140		<2.15	>5.17	30

**Table 6:** Skin irritation scores for female and male New Zealand rabbits following topical application of the PHMB-loaded sol–gel formulation, based on OECD Guidelines No: 404.

Time	Rabbit			
	Female		Male	
	Erythema	Edema	Erythema	Edema
1 h	N.O.	N.O.	N.O.	N.O.
24 h	N.O.	N.O.	N.O.	N.O.
72 h	N.O.	N.O.	N.O.	N.O.

**Table 7:** Three-month stability study of PHMB.

Time	PHMB Concentration (*w*/*v* %)	Comment
0 Month (Baseline)	0.195	Initial concentration
1st Month	0.193	Minor decrease, stable
2nd Month	0.191	Stable
3rd Month	0.189	Stable

## Data Availability

The data that support the findings of this study are available from the authors upon request.
